# Prevalence and Pattern of Congenital Coronary Artery Anomalies in Patients Undergoing Coronary Angiography at a Tertiary Care Hospital of Northern India

**DOI:** 10.7759/cureus.14399

**Published:** 2021-04-10

**Authors:** Jeet Ram Kashyap, Suraj Kumar, Sreenivas Reddy, Raghavendra Rao k, Ojasav Sehrawat, Rashmi Kashyap, Maninder Kansal, Hithesh Reddy, Vikas Kadiyala, Lipi Uppal

**Affiliations:** 1 Cardiology, Government Medical College and Hospital, Chandigarh, Chandigarh, IND; 2 Community Medicine, Dr Yashwant Singh Parmar Government Medical College, Nahan, IND; 3 General Medicine, Government Medical College and Hospital, Chandigarh, Chandigarh, IND

**Keywords:** coronary artery anomalies, anomalous origin, intrinsic anomalies of coronary artery, dual lad, split rca, coronary fistula

## Abstract

Objectives: To evaluate the prevalence and pattern of congenital coronary artery anomalies (CAAs) in the adult population undergoing catheter coronary angiography.

Methods: The coronary angiograms done between October 2015 and September 2020 were reviewed for the presence of coronary anomalies based upon Angelini's classification. The medical record of patients with anomalies was reviewed for symptomatology and indication of angiography.

Results: CAAs were found in 129 (87 males and 42 females) of 6,258 patients giving a prevalence of 2.06%. The mean age was 57.8 ± 11.8 (range 32-81) years. Among these, the anomalous origin and course of the coronaries were the most common anomaly seen in 81 (1.29%) patients, followed by intrinsic anomalies of the coronary arterial system in 44 (0.7%) patients and anomalies of coronary termination and anomalous anastomotic vessels in 2 (0.03%) patients each. Overall, the absence of the left main trunk with a separate origin of the left anterior descending (LAD) and the circumflex artery was the commonest anomaly seen in 46 (0.74%) patients, followed by dual LAD in 35 (0.56%) patients. The anomalous origin of the right coronary artery (RCA) from the left sinus was seen in 14 patients (0.22%) and that of the circumflex artery from the right sinus or right coronary artery was seen in 11 patients (0.17%). The origin of the left main and RCA from ascending aorta was found in eight (0.13%) patients. One (0.02%) patient had a single coronary artery, and another one (0.02%) had all the three coronary arteries arising from the right sinus; however, with separate ostia. The split RCA was seen in nine (0.14%) patients and there were two (0.03%) patients each of coronary artery fistulae, and of anomalous anastomotic vessels.

Conclusions: The prevalence of congenital coronary anomalies in this study was 2.06%. The commonest anomaly was that of origin and courses of the vessels, however, the pattern of anomalies is different from previous studies.

## Introduction

Coronary artery anomalies (CAAs) are rare congenital disorders with varied clinical presentations. Its prevalence among the adult population varies in angiographic and autopsy series and on average is around 1%. In angiographic studies, the incidence ranges from 0.6% to 5.64%, whereas in autopsy series, it is around 0.3% [1,2]. Generally, CAAs are benign in nature and most patients remain asymptomatic, however, some of these may present with various clinical manifestations like angina, dyspnoea, syncope, acute coronary syndrome, heart failure, ventricular arrhythmias and sudden cardiac death (SCD). In fact, CAAs are the second most common cause of SCD in young individuals to hypertrophic cardiomyopathy [3-7]. The classification and nomenclature of CAAs have remained inconsistent, however, the one proposed by Angelini et al. is most commonly followed. According to this classification, the nomenclature is followed as (i) normal if a particular pattern is seen in >1% of the general population; (ii) an anomaly, if any pattern is seen in <1% of the general population. Furthermore, the CAAs are grouped under four subtypes, i.e., anomalies of origin and course, intrinsic anomalies of coronaries, anomalies of termination, and anomalous anastomotic vessels [8-12]. The significance of CAAs is that they may pose diagnostic and therapeutic challenges such as difficulty in engaging coronary ostia or requirement of special catheters and manoeuvres while performing angiography or angioplasty thus resulting in longer fluoroscopic time. Second, the lack of knowledge of anomaly may sometimes lead to accidental damage to these vessels during cardiac surgery. In this work, we present the angiographic prevalence of CCAs among adult patients undergoing coronary angiography at a single tertiary care centre.

## Materials and methods

This single-center, retrospective, observational study was carried out at a tertiary care level hospital of northern India after obtaining the approval of the institutional ethics committee (IEC) of our hospital. The database of our catheterization laboratory stored on Picture Archive and Communication System (PACS) over the last five years (October 2015 and September 2020) was reviewed. All coronary angiograms done during this period were evaluated by two interventional cardiologists independently for the presence of any CAAs. The coronary anomalies were classified as per the Angelini's classification and in case there was any difference in opinion, a senior interventional cardiologist was consulted to reach the consensus. We excluded patients with congenital heart diseases, patients with a separate conal artery, patients with myocardial bridge and post bypass surgery patients.

Statistical methods

Statistical package for social sciences (SPSS software Version 23.0, SPSS, Inc., Chicago, IL, USA) was used for analysis of data. The categorical variables have been presented as percentages (%) and frequencies and continuous variables are presented as mean ± standard deviation (SD).

## Results

A total of 6,258 angiograms were reviewed and we found various CAAs in 129 (2.06%) patients. Among these, there were 87 (67.4%) males and 42 (32.6%) females. The mean age of the patients with CAAs was 57.8 ± 11.8 (range 32-81) years. The highest number, 39 (30.2%), of patients with anomalous coronaries were seen in the age group of 51- 60 years followed by 36 (27.9%) in 61-70 years, 24 (18.6%) in 41-50 years, 15 (11.6%) in 71-80 years and 13 (10.1%) in 31-40 years age group. The indications for undergoing angiography among these patients were acute coronary syndrome in 67 (51.9%), chronic stable angina in 30 (23.2%), atypical chest pain in 20 (15.5%), and in 12 (9.3%) patients it was done as a part of evaluation for left ventricular dysfunction (Table 1).

**Table 1:** Baseline characteristics and pattern of coronary anomalies. All values are presented as mean ± SD or number (%). CAA: coronary artery anomalies.

Characteristics	Numbers
The total number of coronary arteriograms reviewed	6258
Sex distribution (total patients)	
Males	4362 (69.7%)
Females	1896 (30.3%)
Number of patients with CAAs	129 (2.06%)
Anomalies of coronary origin and course	81(1.29%)
Anomalies of intrinsic coronary arterial anatomy	44 (0.7%)
Anomalies of coronary termination	2 (0.03%)
Anomalous anastomotic vessels	2 (0.03%)
Mean age (range) in years of patients with CAAs	57.8 ± 11.8 (32-81)
Gender distribution of patients with CAAs	
No. of male	87 (67.4%)
No. of female	42 (32.6%)
Indication for undergoing coronary angiography	
Acute coronary syndromes	67 (51.9%)
Chronic stable angina	30 (23.2%)
Atypical chest pain	20 (15.5%)
For evaluation of left ventricular dysfunction	12 (9.3%)

The various types of CAAs and their prevalence rates are shown in Table 2.

**Table 2:** Prevalence of various coronary artery anomalies. LAD: left anterior descending, LCS: left coronary sinus, LCx: left coronary circumflex, LMCA: left main coronary artery, RCA: right coronary artery, RCS: right coronary sinus.

Coronary anomaly		No. of patients (n=6258)	Angiographic incidence (%)	Anomaly incidence (%)
Anomalous origin of coronary artery				
	Absent left main trunk with Separate origin of LAD and LCx	46	0.74	35.7
	RCA arising from LCS	14	0.22	10.9
	LCx arising from RCS	7	0.11	5.4
	LCx arising from RCA	4	0.06	3.1
	RCA from ascending Aorta	6	0.10	4.7
	LMCA from ascending aorta	2	0.03	1.6
	Single coronary artery	1	0.02	0.8
	All coronaries from RCS with separate ostia	1	0.02	0.8
Anomalies of intrinsic coronary arterial anatomy				
	Dual LAD	35	0.56	27.1
	Split RCA	9	0.14	7.0
Anomalies of coronary termination				
	Coronary arterial fistulae	2	0.03	1.5
Anomalous anastomotic vessels		2	0.03	1.5
Total		129	2.06	

Anomalies of origin and course

Absence of Left Main Trunk With Separate Origin of LAD and LCx

**Figure 1:**
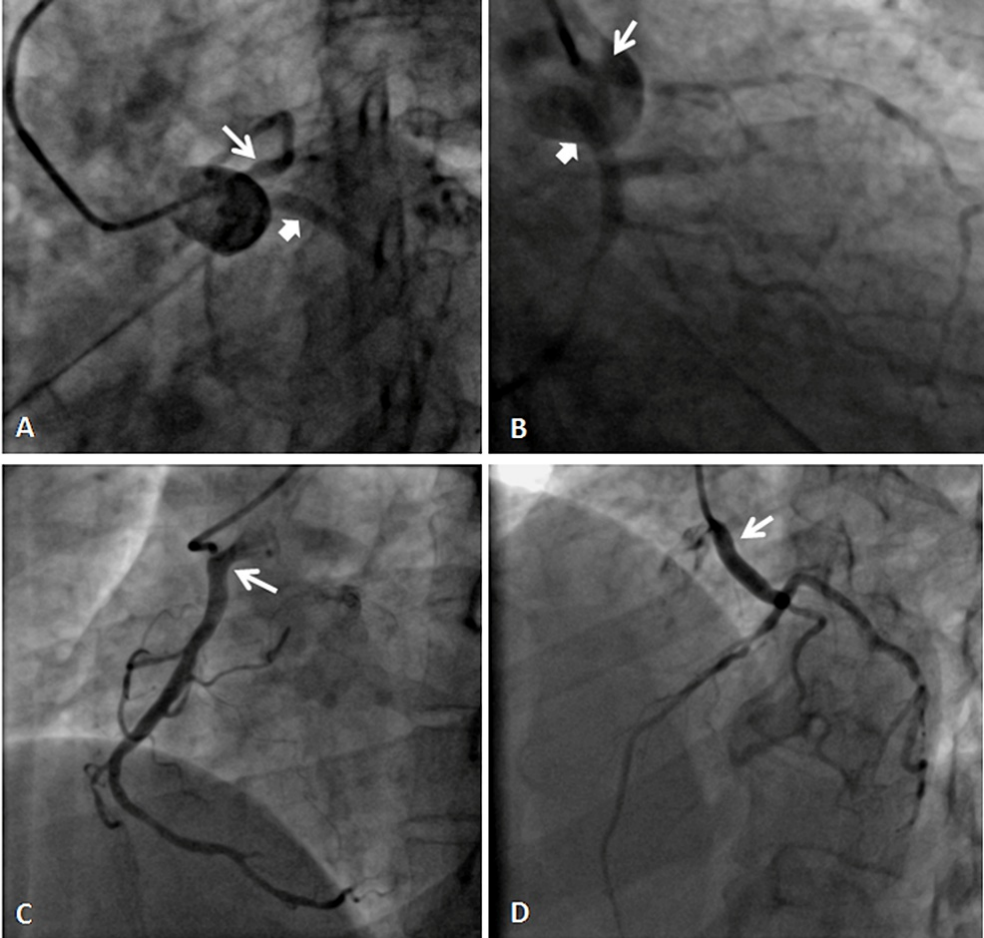
(A) and (B) coronary angiograms showing the separate origin of LAD and LCx from the left sinus, and (C) and (D) show the origin of RCA and LMCA from ascending aorta, respectively. LAD - thin white arrow and LCx - thick white arrow. LAO: left anterior oblique, RAO: right anterior oblique, LAD: left anterior descending, LCx: left coronary circumflex, RCA: right coronary artery, LMCA: left main coronary artery.

The separate origin of LAD and LCx from the left sinus with the absence of the left main trunk was the commonest anomaly seen in 46 (35.7%) patients having an angiographic prevalence of 0.74% (Figure 1A-B and Video 1).

 

**Video 1:** Coronary angiogram in RAO caudal projection showing absent left main trunk with separate origin of LAD artery and LCx from left sinus. RAO: right anterior oblique, LAD: left anterior descending, LCx: left coronary circumflex.

Anomalous Origin of RCA From Left Sinus 

**Figure 2:**
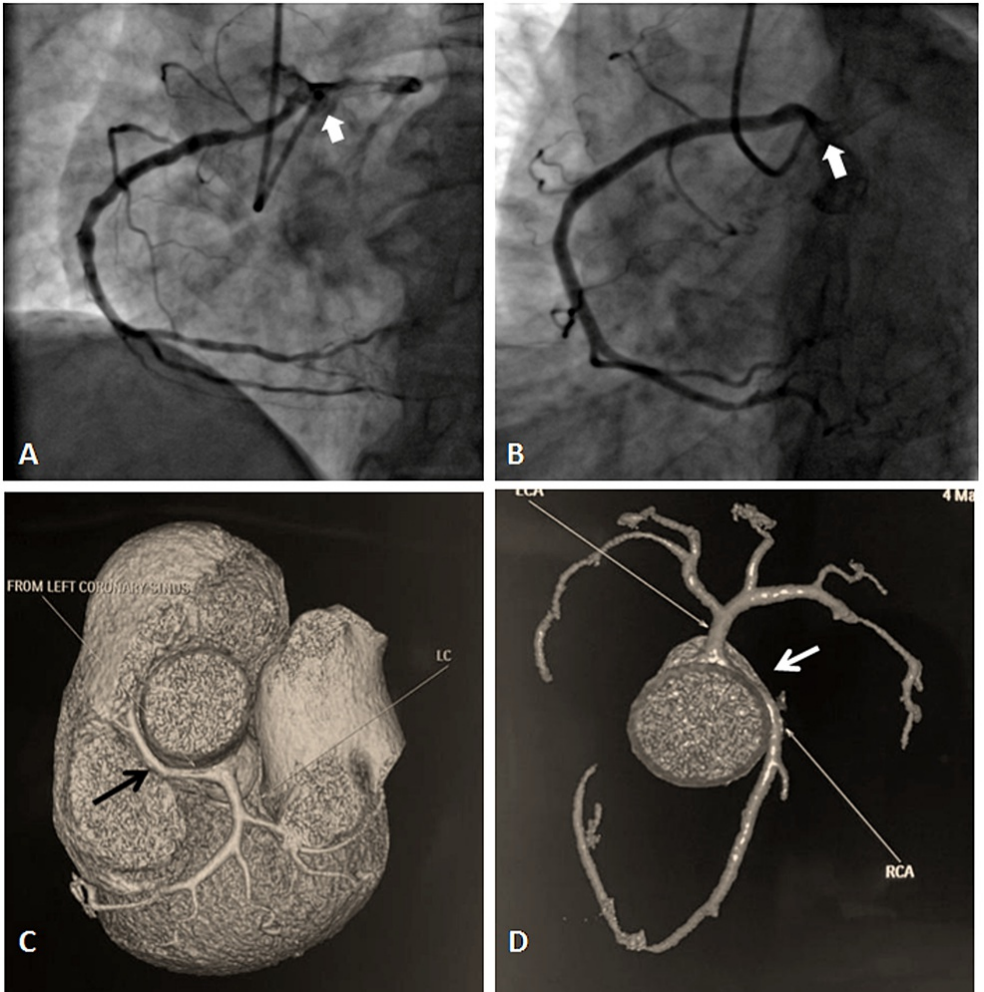
(A) and (B) shows the RCA arising from the left sinus; (C) and (D) CT images demonstrating inter-arterial course anomalous RCA (white arrow). LAO: left anterior oblique, RCA: right coronary artery.

This was the second most common anomaly of origin and course seen in 14 (10.9%) patients with an angiographic prevalence of 0.22% (Figure 2A-D). All patients underwent a successful angiogram using Tiger 5F catheter via the right radial route. However, the catheter had to be pushed deep with some clockwise rotation to selectively engage the vessels. In one of the patients, CT angiography showed a malignant course of RCA traversing between the aorta and pulmonary trunk (Figure 2C-D).

Anomalous Origin of LCx from the RCA/Right Sinus 

**Figure 3:**
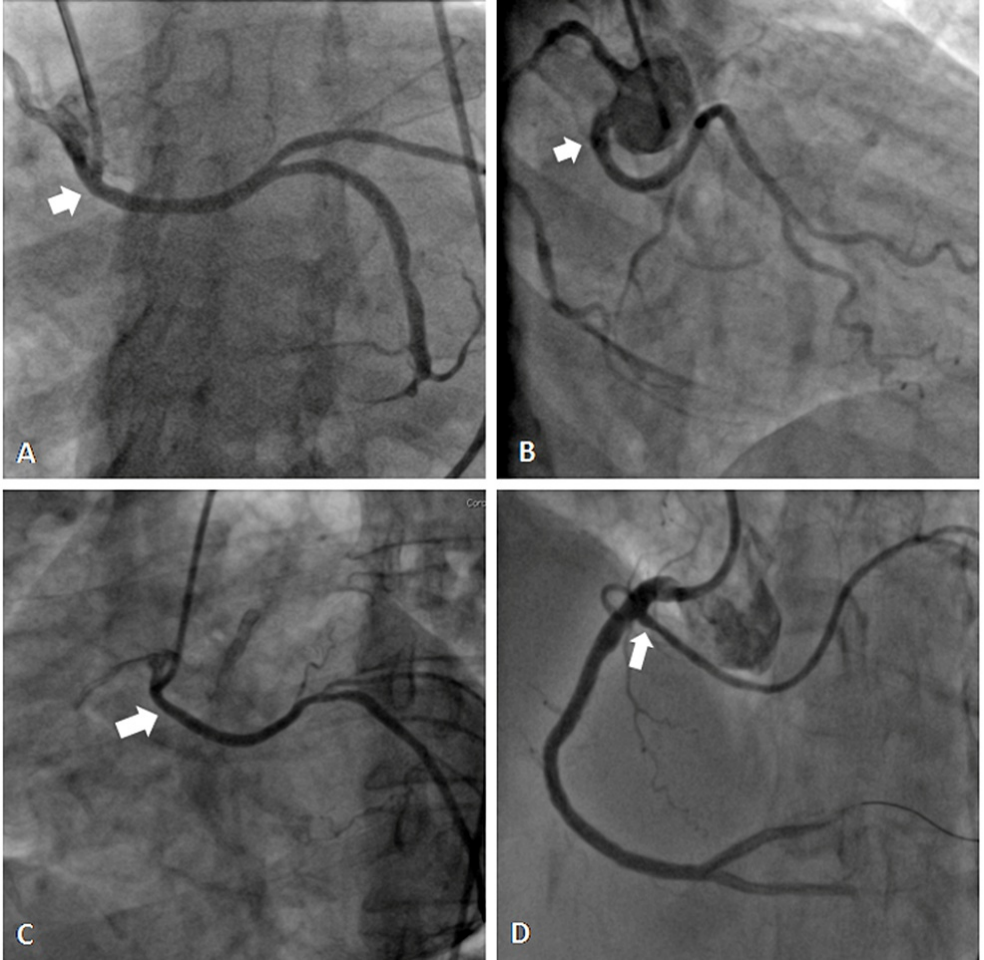
Coronary angiograms showing LCx arising from the right sinus (A-C), and from the right coronary artery (D). LCx: left coronary circumflex.

The LCx originated from the opposite side in 11 patients (7 from right sinus and 4 from RCA), accounting for 8.5% of the anomalies and having an angiographic prevalence of 0.18% (Figure 3A-D and Video 2). In most patients, 10 (90.9%), the angiograms were done by using Tiger 5F catheter and in one (9.1%) patient the Judkin's right (JR-3.5) catheter was used.

**Video 2:** Coronary angiogram showing Cx arising from RCA. LAO: left anterior oblique, Cx: circumflex, RCA: right coronary artery.

Origin of RCA From Ascending Aorta

Right coronary artery originated from ascending aorta in six (4.7%) patients with an angiographic prevalence of 0.1% (Figure 1C and Video 3). The RCA was selectively engaged with Tiger 5F catheter in two patients and Amplatzer's Left (AL1) in two patients. However, in two patients the RCA could not be hooked selectively and the ascending root aortogram using 6F Pigtail was performed to visualize the anomalous RCA.

**Video 3:** Aortogram showing right coronary artery arising from ascending aorta.

Origin of LMCA From Ascending Aorta

The left main coronary artery (LMCA) was seen originating from ascending aorta in two (1.6%) patients having a prevalence of 0.02% (Figure 1D and Video 4). The angiogram was done using a Tiger 5F catheter.

**Video 4:** Coronary angiogram in left anterior oblique projection showing the origin of the left main coronary artery from ascending aorta.

Single Coronary Artery

**Figure 4:**
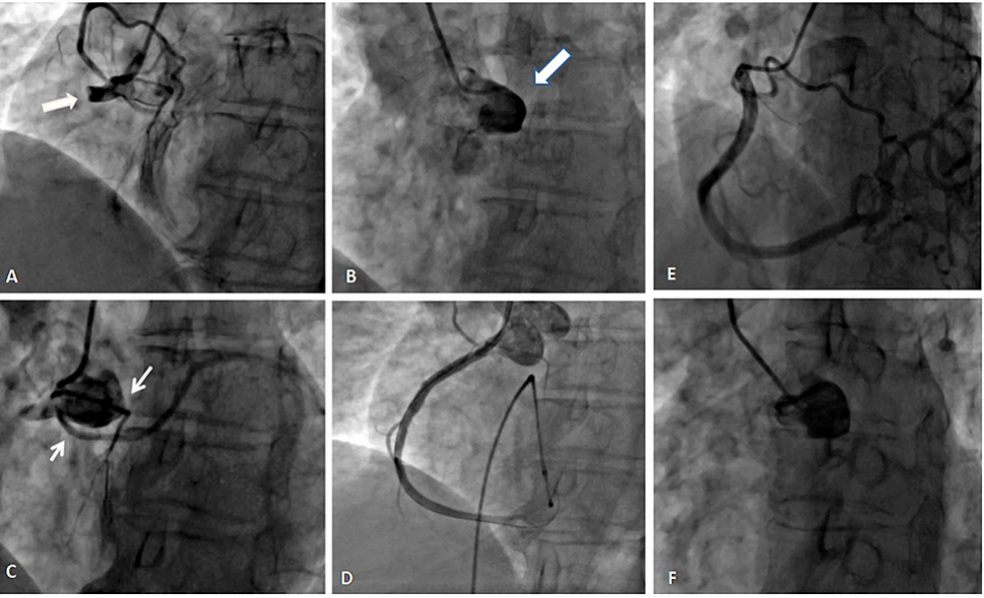
(A-D) Coronary angiogram showing all coronaries arising from the right sinus; (E-F) showing single coronary artery (E) and bare left sinus (F). (A) Coronary angiogram in LAO projection showing all coronaries arising from the right sinus with totally occluded RCA; (B) shows bare left sinus; (C) shows LAD (white arrow) and LCx (white arrow) arising from the right sinus with separate ostia's; (D) large (RCA) after primary angioplasty. LAO: left anterior oblique, LCx: left coronary circumflex, LAD: left anterior descending, RCA: right coronary artery.

In one (0.8%) patient, only a single coronary artery was seen arising from the right sinus and supplying the whole myocardium (Figure 4 and Video 5). Although, the patient underwent angiography with the suspicion of coronary artery disease, however, there was no involvement by atherosclerosis of any branch of this single coronary artery.

**Video 5:** Coronary angiogram in right anterior oblique projection showing single coronary artery.

All Coronaries From Right Sinus

In one (0.8%) of the patient, all the three coronaries were seen arising from the right sinus, however, with separate ostia's (Figure 4 and Video 6). The selective injection was done with Tiger 5F catheter with slight clockwise and anticlockwise rotation of the catheter. This patient had presented to us with acute myocardial infarction of the inferior wall and cardiogenic shock. The left shoot showed a bare sinus (Figure 4). The right coronary angiogram showed a large RCA with total thrombotic occlusion in the proximal part. Slight rotation of diagnostic catheter showed LCx and small LAD arising from the same sinus with separate ostia. The primary PCI with stenting to RCA was done using Judkin’s Right (JR3.5) guide catheter.

**Video 6:** Coronary angiogram in left anterior oblique projection showing all coronaries arising from the right sinus.

Anomalies of intrinsic coronary arterial anatomy

**Figure 5:**
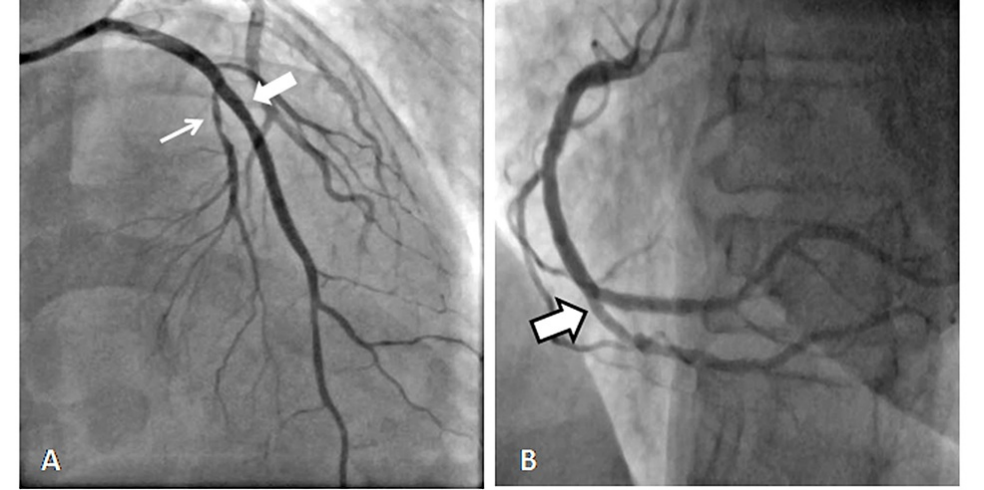
(A) Coronary angiogram showing dual left anterior descending and (B) showing split right coronary artery.

Dual LAD (Figure 5A) was seen in 35 (27.1%) patients, with an angiographic prevalence of 0.84%. The atherosclerotic involvement was seen in 25 patients. The split RCA was seen in nine (7%) patients with an angiographic prevalence of 0.14% (Figure 5B).

Anomalies of coronary termination

**Figure 6:**
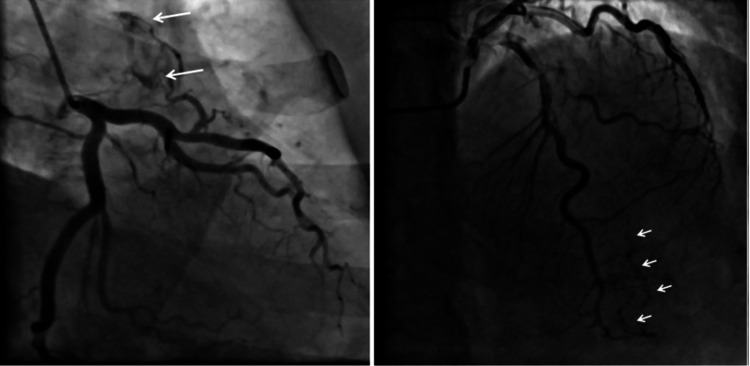
Coronary angiogram in RAO caudal projection showing the fistula from a small branch of diagonal (D1) and to left pulmonary artery (A). (B) AP cranial projection showing multiple fistulae from distal LAD, with termination into the right ventricle. RAO: right anterior oblique, LAD: left anterior descending.

Coronary cavernous fistulae were seen in two (1.5%) patients, with an angiographic prevalence of 0.03%. In one patient, the fistula was arising from a small branch of diagonal (D1) and draining into the left pulmonary artery (Figure 6A). The other patients had small multiple fistulae from distal LAD, with termination into the right ventricle (Figure 6B).

Since these fistulas were very small in both the patients, no intervention was done.

Anomalous anastomotic vessels

**Figure 7:**
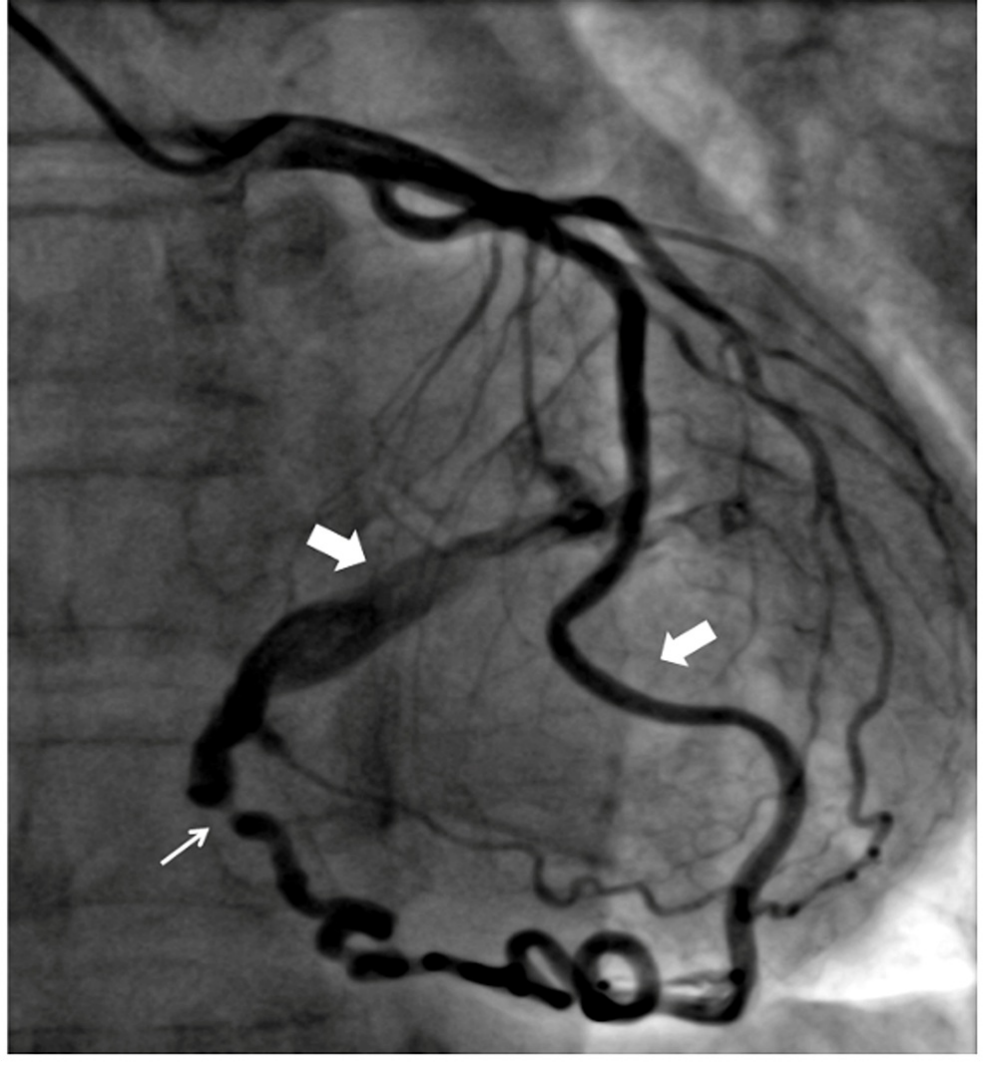
Coronary angiogram showing anomalous anastomotic channel arising from distal LAD and draining into left atria with a stenotic segment just before termination (white arrow). LAD: left anterior descending.

Anomalous anastomotic vessels were found in two (1.5%) patients. One patient had a long tortuous channel arising from terminal LAD with termination into the left atria (Figure 7 and Video 7).

**Video 7:** Coronary angiogram in RAO projection showing anomalous anastomotic channel arising from distal LAD, draining into left atria, and having stenosis before termination. RAO: right anterior oblique, LAD: left anterior descending.

**Figure 8:**
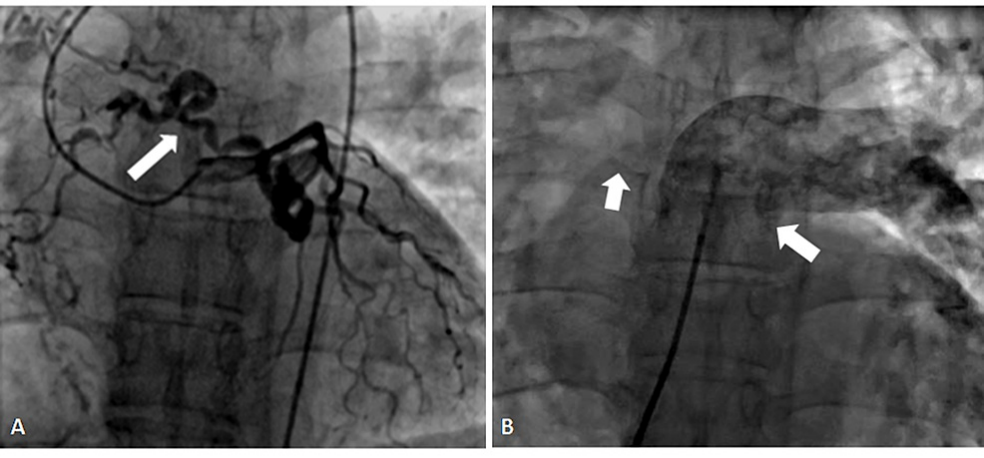
Coronary angiogram showing collaterals arising from LCx artery supplying the right lung (A) and (B); pulmonary angiogram showing absent right pulmonary artery. LCx: left coronary circumflex, RPA: right pulmonary artery.

This patient had class II angina which improved with standard antianginal therapy. The channel had stenosis in the midpart, hence no intervention was done. The second patient had a long collateral channel arising from distal LCx and supplying the hilar region of the right lung. In this patient, the right pulmonary artery (RPA) was absent and the systemic collaterals were seen from the right subclavian artery and also from LCx. This patient presented with atypical chest pain and stress thallium showed a <5% perfusion defect in LAD territory. He was managed conservatively and is doing well (Figure 8A-B and Videos 8-9).

**Video 8:** Coronary angiogram in anterior-posterior caudal projection showing collaterals arising from the left circumflex artery supplying the right lung.

**Video 9:** Pulmonary angiogram showing absent right pulmonary artery from the main pulmonary artery.

## Discussion

Coronary artery anomalies are a very rare form of congenital anomalies. The classification of these anomalies is quite varied and the one proposed by Angelini is widely followed. This classification describes coronary anomalies under four subtypes, out of which the anomalies of origin and course appear clinically most significant in terms of challenges faced during angiography and surgery [13]. The anomalous origin especially those with the malignant course are responsible for symptoms and sometimes sudden cardiac death and moreover, they may pose problems during surgery like accidental ligation or damage.

The overall prevalence of CAAs in our study was 2.06% which is in the range of other studies (0.6-5.64%) reported by various authors [1,8,10,14-17]. The study with the largest angiographic population [1] had an incidence of 1.3%, while the study with a prospective design using strict diagnostic criteria [8] had the highest incidence of 5.64%. We also followed Angelini's classification, and the prevalence of anomalies of origin and course in our study was highest at 1.29% followed by anomalies of intrinsic coronary arterial anatomy (0.7%). This is, however, in contrast to Sidhu et al. who reported these anomalies in reverse to us, i.e., in 1.33% and 1.52%, respectively [18]. This may be because of the smaller sample size in their study and a different geographic location. In our study, the prevalence of anomalous coronaries was higher among males almost double (2.07:1 male to female ratio). Similar observations have been made by other authors where the male predominance is seen up to the tune of 3:1 ratio [18-20]. The reason for this higher prevalence among male patients could be the fact that a greater number of male patients as such underwent angiograms (4362 males and 1896 females among 6258 patients) in general largely because of the fact that male patients have a higher prevalence of coronary artery disease. Another reason could be the gender bias for the lower overall number of female patients undergoing angiography, which is quite prevalent in our country.

The most common anomaly was the separate origin of LAD and LCx from the left sinus without any left main trunk. The angiographic prevalence of 0.74% in our study was higher than the previously reported data by Yamanaka and Hobbs (0.37%) [1], Sohrabi et al. (0.69%) [19], Harikrishnan et al. (0.16%) [21], and Nawale et al. (0.45%) [20]. The probable reason is that instead of analysing only angiographic reports, we reviewed all 6258 angiographic films and discussed them with experienced interventionists. As such this anomaly does not cause any haemodynamic impairment and therefore, considered benign, however, we may have to use special manoeuvres or catheters while engaging these separately arising coronaries. The next common anomaly was dual LAD seen in 27% with an angiographic prevalence of 0.84% of patients which is similar to that reported by Spindola-Franco et al. [22] and Sidhu et al [18].

The RCA arising from the left sinus had an angiographic prevalence of 0.22% which is nearly similar to Garg et al. (0.37%), although in their study this was the commonest anomaly in contrast to ours [10]. Other studies had lower prevalence like in a study by Sohrabi et al. the prevalence was (0.1%) [19] and Harikrishnan et al. it was (0.1%) again [21]. The clinical significance of this anomaly lies in the fact that it may be difficult to engage this anomalous vessel because of the slit-like orifice and requirement of a special catheter. Second, the malignant course, i.e., between aorta and pulmonary trunk may lead to myocardial ischemia during exertion and sometimes even sudden cardiac death. The malignant course was seen in one of our patients in whom the sole reason for angina and undergoing coronary angiography was the inter-arterial course of anomalous RCA confirmed by CT angiography. Since this was a retrospective study so the CT angiography data were not available in all patients. However, we are of the opinion that CT angiography should be done in all patients with anomalous origin of the coronary artery from opposite sinus to know the exact course of the vessel.

The LCx may arise from the RCS or from the RCA. In our study, the prevalence of LCx arising from RCS/RCA was 0.17% which is lower when compared to other studies, Sohrabi et al. (0.28%) [19], Garg et al. (0.34%) [10], and Nawale et al. (0.33%) [20]. On angiography, this type of anomaly is usually suspected when the left main trunk is long and definite LCx coursing in the AV groove is not seen. This anomaly is usually benign, however, carries the risk of accidental ligation or injury during valve surgery. Similarly, the anomalous origin of the LCA or RCA from ascending aorta is usually benign but runs the risk of accidental cross-clamping or complete transection during surgery if the surgeon is not aware of this anomaly [23]. The angiographic prevalence of anomalous RCA arising from ascending aorta of 0.1% was similar to that seen by Yamanaka and Hobbs et al. (0.15%) [1] and Sidhu et al. (0.12%) [18]. We also had two cases of left main coronary trunk arising from ascending aorta. This was quite less as compared to Yamanaka et al. [1] who has reported 16 such cases in their study. The reason for this may be the different sample sizes and different geographical locations.

We had a single case (0.8%) of a single coronary artery supplying the whole myocardium. This is a very rare anomaly and in some of the patients can cause sudden cardiac death in whom the major branch runs between the aorta and main pulmonary trunk [24]. In Yamanaka's series, there were 56 cases of single coronary artery out of a total of 1,26,595 patients [1]. However, the number of cases reported from other parts of the Indian subcontinent is very few like in our study. Harikrishnan et al. [21] reported three cases in a series of 7,400 patients, Lingaraju et al. [25] reported two cases, and Nawale et al. [20] only one case. 

One of our patients had a very rare anomaly of all the three coronaries originating from the right sinus. This rare anomaly was seen by Harikrishnan et al. [21] and Sohrabi et al. [19] in one patient each. This patient had thrombotic total occlusion of RCA. The patient underwent primary PCI to RCA, however, succumbed later due to multi-organ failure. The significance of this anomaly becomes greater in a situation like in our patient, in which the major vessel had acute total thrombotic occlusion and the patient could not tolerate the guide catheter even for few minutes because of very close ostia's of smaller vessels and had a significant drop in blood pressure. Therefore, the percutaneous intervention in this setting becomes high risk and care should be taken to avoid deep engagement and preferably keeping guide catheter outside the ostia.

The anomalies of termination and anomalous anastomotic vessels were the least common with an angiographic prevalence of 0.06%, which was similar to previous studies [18,26]. One patient had anomalous anastomotic channels from the left circumflex artery supplying the right lung with an absent RPA. This type of configuration has been described in a limited number of case reports previously [27,28].

Our study had the following limitations. The study population was the patients undergoing angiography for acute coronary syndrome or chronic stable, which as such does not represent the general population. Therefore, this is not the true representation of the prevalence of coronary anomaly in the general population. Similarly, being a single-centre study also does not reflect the overall true prevalence in the general population. The other modalities like CT or magnetic resonance coronary angiography were done in a limited number of patients only so the course and malignant nature could not be defined in all patients. A prospective study including multidetector CT will be more informative.

## Conclusions

The prevalence of CAAs in the present study was 2.06%. The anomalies of origin and course were most common, but patterns of anomalies in the present study were different from previous studies. Overall, the absence of the left main trunk with separate origin of LAD and LCx was the most common anomaly followed by dual LAD. Angiographic recognition of CAAs is important because of their clinical significance and importance in patients undergoing coronary angioplasty or cardiac surgery. CT coronary angiography was done in a limited number of patients in this study and we are of the opinion that it should ideally be done in all patients with anomalous origin of the coronary artery from opposite sinus to know the exact course of the vessel.
